# Decoding the immune microenvironment in osteosarcoma: new insights into checkpoints, vaccines, and CAR-T cells

**DOI:** 10.3389/fonc.2025.1702219

**Published:** 2025-10-31

**Authors:** Haitian Wang, Tongtong Zhu, Kunpeng Yang, Chenkai Xu, Zhaoyi Zhang, Guangyao Liu, Hongge Ren

**Affiliations:** ^1^ Department of Traumatic Orthopedics, China-Japan Union Hospital of Jilin University, Changchun, China; ^2^ School of Clinical Medicine, Changchun University of Chinese Medicine, Changchun, China; ^3^ Department of Physiology and Pathophysiology, College of Medicine, Yanbian University, Yanbian, China; ^4^ Department of Joint Surgery, Yanbian University Hospital, Yanbian, China

**Keywords:** osteosarcoma, tumor microenvironment, immunosuppression, immune checkpoint inhibitors, immunotherapy, combinatorial approaches

## Abstract

Osteosarcoma (OS), the most prevalent primary malignant bone tumor, disproportionately affects adolescents and is marked by rapid progression and a high rate of pulmonary metastasis. Despite advances in multimodal treatment, outcomes remain dismal for metastatic or relapsed disease, largely due to chemoresistance, immune evasion, and the heterogeneous tumor microenvironment (TME). Increasing evidence underscores the immunoregulatory complexity of osteosarcoma, characterized by immunosuppressive myeloid-derived populations, dysfunctional lymphocyte infiltration, and exosome-mediated immune escape. While immune checkpoint inhibitors have revolutionized treatment in several malignancies, their impact in osteosarcoma remains modest, highlighting the need for combinatorial strategies. Emerging approaches such as adoptive T cell therapies, tumor vaccines, and CAR-T cell interventions are being explored to overcome the “cold” immune milieu. Furthermore, single-cell transcriptomics has shed light on cellular interactions within the osteosarcoma TME, offering insights into resistance mechanisms and potential biomarkers. This review provides a comprehensive overview of the immunological landscape of osteosarcoma and highlights current and emerging immunotherapeutic strategies aimed at improving clinical outcomes in this challenging malignancy.

## 1 Introduction

Osteosarcoma represents the most prevalent primary malignant tumor of bone, with a marked predilection for adolescents and young adults (1, 2). It arises predominantly within the metaphyseal regions of long bones, notably the distal femur, proximal tibia, and humerus. Clinically, osteosarcoma follows an aggressive trajectory, with subclinical pulmonary micrometastases observed in approximately 80–90% of patients at initial diagnosis. Molecularly, it is characterized by profound heterogeneity across genomic, epigenomic, and transcriptomic layers. Frequent molecular aberrations include inactivation of key tumor suppressors such as TP53 and RB1, disruptions in mesenchymal differentiation pathways, and extensive epigenetic reprogramming mediated by inflammatory signals. These alterations collectively compromise apoptotic control and perturb skeletal tissue homeostasis. Notably, epigenetic modulators like histone modification enzymes and DNA methyltransferases dynamically reshape gene expression profiles, particularly those involving regulatory non-coding RNAs, thereby accelerating tumor progression and correlating with adverse clinical outcomes (3).

The current therapeutic standard comprises radical surgical resection in combination with multi-agent chemotherapy, most commonly employing methotrexate, doxorubicin, and cisplatin (MAP regimen). Although targeted agents such as tyrosine kinase inhibitors (sorafenib, apatinib) have been evaluated, their clinical benefit remains inconsistent (4). Persistent challenges, including chemoresistance, immune evasion, and intratumoral heterogeneity, continue to hinder therapeutic efficacy (5). In response, research has increasingly focused on immunomodulatory strategies. Preclinical and clinical studies underscore the promise of immunotherapy, with evidence including lipopolysaccharide-induced tumor regression and prolonged survival following mifamurtide administration, substantiating the immunogenic nature of osteosarcoma (6–8). Nonetheless, the profoundly immunosuppressive tumor microenvironment poses a significant barrier. Ongoing investigations into immune checkpoint blockade, adoptive T cell therapies, cancer vaccines, and combinatorial immunotherapeutic regimens offer a path forward, with the potential to redefine treatment paradigms for this recalcitrant malignancy.

## 2 Immune landscape in osteosarcoma

The tumor microenvironment (TME) exerts profound influence over the initiation, progression, and metastatic potential of osteosarcoma (9, 10). Comprising a complex interplay between bone marrow stromal elements and an extensive vascular network, the TME provides a permissive niche that supports tumor cell survival, proliferation, and immune evasion by modulating osteogenic and immunological interactions (11, 12). Notably, osteosarcoma is heavily infiltrated by diverse immune cell subsets, including macrophages, dendritic cells, neutrophils, natural killer (NK) cells, and lymphocytes, reflecting a dynamic immunological landscape (13). Within this complex immunological milieu, exosomes play pivotal roles in promoting tumor growth, metastasis, and therapy resistance via immune modulation (14, 15). Mechanistically, these vesicles promote immunosuppression by inducing T cell apoptosis, impairing cytotoxic T and NK cell function, and expanding populations of myeloid-derived suppressor cells (MDSCs) (16). Analogous mechanisms observed in prostate cancer, wherein tumor-derived exosomes downregulate NKG2D expression on CD8^+^ T cells and NK cells, underscore the conserved role of exosomes in dampening cytotoxic immunity (17, 18). In osteosarcoma specifically, exosome-associated TGF-β2 has been shown to skew macrophage polarization toward an M2-like phenotype, thereby enhancing tumor invasiveness (19, 20). Furthermore, PD-L1–bearing exosomes correlate with pulmonary metastasis and hold promise as non-invasive prognostic biomarkers (21). Exosomal delivery of indoleamine 2,3-dioxygenase (IDO) further contributes to immune evasion by disrupting tryptophan metabolism and fostering an angiogenic microenvironment (22, 23).

**Figure 1:**
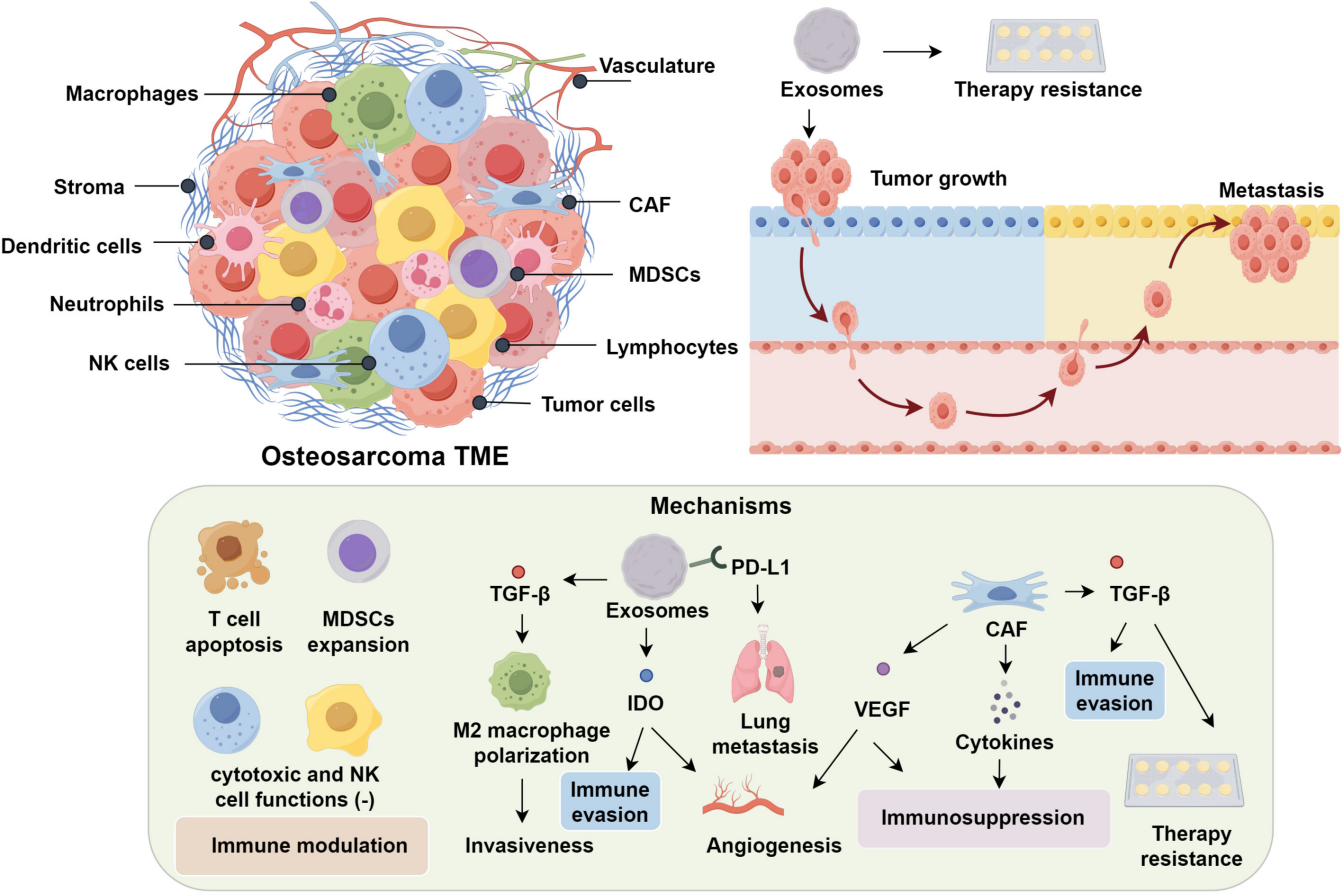
Immune Microenvironment and Immunotherapy in OS.

Cancer-associated fibroblasts (CAFs) contribute to the establishment of an immunosuppressive tumor microenvironment through the secretion of tumor-promoting cytokines, thereby supporting immune evasion and disease progression (24, 25). TGF-β has emerged as a pivotal orchestrator of immune escape and therapeutic resistance in osteosarcoma. Dual blockade of TGF-β signaling, particularly when combined with dendritic cell–based immunotherapeutic approaches, demonstrates synergistic antitumor activity in preclinical models (26–28). In parallel, VEGF facilitates neovascularization while concurrently promoting immunosuppression. Pharmacological inhibition of VEGF using multi-kinase inhibitors such as sunitinib not only impairs angiogenesis but also attenuates MDSC accumulation and enhances intratumoral infiltration of CD8^+^ cytotoxic T lymphocytes (29–31). Beyond its anti-angiogenic capacity, sunitinib has been shown to modulate the immune landscape by depleting MDSCs and enabling effector T cell infiltration (32). Preclinical models have shown that sunitinib-mediated VEGF blockade reconditions the immunosuppressive niche, thereby enhancing CD8^+^ T cell trafficking and function—effects that provide a compelling rationale for combinatorial strategies with immune checkpoint inhibitors (33). Supporting this, interim results from the NCT03277924 clinical trial revealed that co-administration of sunitinib with the PD-1 inhibitor nivolumab achieved disease stabilization in nearly 50% of patients with advanced sarcomas (34). Tumor-associated macrophages (TAMs), another dominant immune component within the osteosarcoma microenvironment, exhibit marked functional plasticity. While M1-polarized TAMs mediate antitumor immunity, their M2-like counterparts facilitate metastasis through upregulation of COX-2, MMP9, phosphorylated STAT3, and epithelial–mesenchymal transition (EMT)–related markers (35, 36). Transcriptomic analyses reveal that M2-skewed signatures correlate strongly with pulmonary metastatic potential. Pharmacological reprogramming of TAMs toward an M1 phenotype, such as via all-trans retinoic acid, has demonstrated potent antimetastatic effects (20, 37). These findings highlight the therapeutic promise of TAM repolarization, as M2-dominant profiles are consistently linked with heightened metastatic burden, while reprogramming TAMs toward M1 reduces metastatic burden (38) (**Figure 1**).

## 3 Immunotherapeutic approaches in osteosarcoma

### 3.1 Immune checkpoint inhibition in osteosarcoma

Recent progress in cancer immunotherapy underscores the therapeutic promise of immune checkpoint blockade, particularly targeting CTLA-4, B7-H3, PD-1, and PD-L1. Approximately 23.7% of osteosarcoma specimens exhibit high PD-L1 expression, with moderate expression levels observed in nearly 50% of cases—patterns that are positively associated with metastasis, tumor-infiltrating lymphocytes, and reduced five-year survival rates (39). Notably, PD-L1 expression is more prevalent in metastatic pediatric osteosarcoma than in localized disease (40). A cohort study involving 234 patients further revealed that positivity for PD-L1 and PD-1 significantly correlated with poor prognosis and decreased overall survival (41). Nevertheless, clinical trials evaluating immune checkpoint inhibitors in osteosarcoma have yielded underwhelming results. In the SARC028 trial, pembrolizumab elicited a modest 5% objective response rate (42), a finding corroborated by the NCT03013127 study (43). Similarly, pediatric trials investigating nivolumab (NCT02304458) and atezolizumab (NCT02541604) failed to demonstrate meaningful clinical benefit. These disappointing outcomes are thought to reflect the immunologically inert tumor microenvironment of osteosarcoma, characterized by limited T cell infiltration and effector function (44). In contrast, preclinical models suggest that PD-1 blockade can partially restore T cell functionality under experimental conditions (45). In humanized mouse models, nivolumab has been shown to suppress metastatic spread by enhancing the activation of CD4^+^ and CD8^+^ T cells and promoting M1 macrophage polarization. However, such immune activation is often insufficient in isolation, particularly in tumors with low baseline immunogenicity (46). Combination strategies may offer a more promising therapeutic avenue. Dual blockade of PD-1 and CTLA-4 in murine K7M2 models achieved superior tumor control and increased intratumoral CD8^+^ T cell infiltration compared to monotherapy (46, 47). These preclinical findings have been mirrored in clinical settings. The Alliance A091401 trial demonstrated a 16% objective response rate in patients receiving combined nivolumab–ipilimumab therapy, compared to 5% with nivolumab alone, underscoring the potential of dual checkpoint inhibition in overcoming immunotherapy resistance in osteosarcoma (48) (**Table 1**).

**Table 1:** Immunosuppressive mechanisms in TME of the OS.

Component	Mechanism of immunosuppression	Molecular mediators	Clinical values
Tumor-Associated Macrophages (TAMs)	M2 polarization promotes metastasis, EMT, angiogenesis	TGF-β, IL-10, COX-2, MMP9, p-STAT3	High M2:M1 ratio correlates with poor prognosis; reprogramming strategies under evaluation
Myeloid-Derived Suppressor Cells (MDSCs)	Inhibit T cell activation and proliferation	ARG1, ROS, IL-10, PD-L1	Promote immune evasion; targeting via anti-VEGF, all-trans retinoic acid
Cancer-Associated Fibroblasts (CAFs)	Remodel ECM, promote immune cell exclusion	TGF-β, VEGF, CXCL12	Enhance immunosuppressive niche; potential synergy with anti-TGF-β agents
Exosomes	Deliver immunosuppressive signals, inhibit cytotoxic cells	PD-L1, IDO, TGF-β2, miRNAs	Elevated exosomal PD-L1 in metastatic osteosarcoma; potential diagnostic/prognostic marker
T Cells (CD8^+^, CD4^+^)	Exhaustion and reduced cytotoxicity	PD-1, TIM-3, LAG-3, CTLA-4	Limited tumor infiltration; checkpoint blockade strategies ongoing
Dendritic Cells (DCs)	Tolerogenic phenotype reduces antigen presentation	IL-10, IDO, STAT3 activation	DC-based vaccines under development to restore antigen presentation
Immune Checkpoints	Direct inhibition of T/NK cell function	PD-1/PD-L1, CTLA-4, B7-H3	Key targets for ICIs; combination therapies being explored
Tryptophan Metabolism	Immune tolerance via metabolite-induced suppression	IDO1, TDO2, kynurenine	Associated with poor response to ICIs; dual inhibitors in preclinical studies

### 3.2 Emerging immunotherapeutic targets and resistance mechanisms

B7-H3, an immune checkpoint molecule, is markedly overexpressed in osteosarcoma and various malignancies, while minimally expressed in normal tissues (26, 49). It exerts immunosuppressive functions by delivering inhibitory costimulatory signals that dampen T cell proliferation and cytokine secretion. High B7-H3 levels are associated with increased tumor invasiveness, recurrence, and poor prognosis. Notably, silencing B7-H3 impairs lymphoma progression, and dual blockade of PD-1 and B7-H3 enhances antitumor immunity (50–52). Ongoing clinical trials for B7-H3 chimeric antigen receptor (CAR)-modified T cells therapy in solid tumors including NCT04897321, NCT04483778, NCT04670068 (53). B7-H3-specific CAR-T cells demonstrated significant anti-tumor activity in models of osteosarcoma, as evidenced by both *in vitro* and *in vivo* experiments. These findings indicate the potential therapeutic utility of B7-H3-directed CAR-T cell immunotherapy in the management of osteosarcoma (54). Besides, B7-H3-CXCR2 CAR T cells significantly improve the anti-tumor activity in osteosarcoma (55). Another pivotal checkpoint, CTLA-4, a type I transmembrane glycoprotein predominantly expressed on regulatory and memory T lymphocytes, acts by outcompeting CD28 for binding to the B7 ligands CD80 and CD86 on antigen-presenting cells, thereby dampening co-stimulatory activation (56). The CTLA-4-targeted monoclonal antibody ipilimumab is the first second-generation immune checkpoint inhibitor sanctioned for melanoma treatment (57). In osteosarcoma, accumulating data suggest a link between heightened CTLA-4 expression and disease progression (58), potentially mediated through mechanisms involving IDO induction, reduced T cell proliferation, and altered inflammatory cytokine signaling.

CTLA-4 inhibition revitalizes anti-tumor immunity by interrupting the B7–CD28 costimulatory axis and promoting the depletion of immunosuppressive regulatory T cells. In a phase I trial, Merchant et al. reported disease stabilization in approximately 25% of pediatric osteosarcoma cases treated with ipilimumab. Although immune-related toxicities were generally tolerable, gastrointestinal complications were more frequently observed in younger patients (59). Despite these advances, immune checkpoint blockade shows limited efficacy in osteosarcoma. Barriers include low PD-L1 expression, scarcity of tumor-specific neoantigens, inadequate cytotoxic lymphocyte infiltration, and a dense desmoplastic stroma (60). The osteosarcoma extracellular matrix (ECM), composed of collagen I, fibronectin, and hyaluronic acid, restricts T cell penetration, This leads to immune cell sequestration at tumor margins, away from PD-L1-enriched regions (61). Moreover, CAF-derived TGF-β enhances checkpoint molecule expression (PD-1, TIM-3) and suppresses T cell cytotoxicity (62). To overcome these challenges, combinatorial strategies integrating immune checkpoint inhibitors with immunoenhancing modalities, such as anti-angiogenic agents, chemotherapy, or radiotherapy, are being actively explored. Future directions include the identification and targeting of novel checkpoint pathways beyond PD-1 and CTLA-4, expansion of patient eligibility criteria in clinical trials, elucidation of resistance-associated molecular pathways, translation of preclinical insights into human studies, and the development of predictive biomarkers to guide treatment selection. Addressing these priorities is critical for the advancement of safer and more effective immunotherapies.

### 3.3 Tumor-infiltrating lymphocyte therapy

Tumor-infiltrating lymphocytes (TILs), primarily composed of T cells and NK cells, play a pivotal role in antitumor immunity through granule-mediated cytotoxicity and immune cascade activation (63). Notably, their specificity for tumor-restricted neoantigens permits targeted cytolysis with minimal off-target effects (64). In osteosarcoma, however, TIL presence is highly heterogeneous, ranging from immune-desert to immune-excluded phenotypes (65, 66). Even in infiltrated tumors, chronic antigen stimulation and immunosuppressive cues can drive T cell exhaustion, undermining responses to monotherapies such as PD-1 blockade (67). To overcome these limitations, adoptive TIL therapy, extraction, ex vivo expansion, and reinfusion of autologous lymphocytes, is under clinical exploration. While isolating functional TILs from osteosarcoma tissue is technically challenging (68, 69), preclinical evidence confirms that expanded TILs retain tumor-homing and cytolytic capabilities against allogeneic osteosarcoma cells (70). Encouragingly, the TIL product lifileucel (LN-144) demonstrated durable disease stabilization in 80% of advanced melanoma patients refractory to checkpoint inhibitors (71), prompting clinical investigation of LN-145 in osteosarcoma (NCT03449108). Initial outcomes reveal prolonged disease control and improved survival, reinforcing TILs’ translational potential in osteosarcoma. The efficacy of TIL therapy is further enhanced by combination with immune checkpoint inhibitors. CTLA-4 blockade has been shown to augment HLA binding affinity and stimulate CD8^+^ T cell proliferation in murine models (72). In osteosarcoma, Wang et al. reported that combining TILs with PD-1 inhibitors significantly improved objective response rates and prolonged both progression-free and overall survival compared to PD-1 monotherapy (73). Similarly, a study involving 60 patients with chemotherapy-refractory metastatic osteosarcoma demonstrated the feasibility and clinical activity of combined TIL/PD-1 therapy (74). Mechanistically, checkpoint blockade revitalizes exhausted CD8^+^ TILs by disrupting immunosuppressive signaling (75). Persistent antigen exposure induces expression of inhibitory receptors such as PD-1, TIM-3, and LAG-3, impairing cytokine production and effector function (76). PD-1 blockade reactivates T cells by restoring TCR signaling and reversing exhaustion-associated transcriptional programs (TOX, NFAT) (77, 78). Although the optimal treatment paradigm, monotherapy versus combination, is yet to be fully established, accumulating evidence suggests that TILs can circumvent resistance and potentiate antitumor immunity in osteosarcoma.

### 3.4 Tumor vaccine

Cancer vaccination strategies aim to elicit tumor-specific immune activation by introducing tumor-associated antigens (TAAs) through platforms such as whole-cell lysates, subcellular fractions, recombinant proteins, or nucleic acid-based vectors. These modalities are often designed to potentiate antigen-specific responses, frequently in conjunction with monoclonal antibodies that recognize tumor surface markers (79). Early work by Marcove et al. (80) demonstrated that immunization using autologous tumor lysates was associated with improved overall survival. Building on this, Mason and colleagues (81) developed DXS31-164, a HER2/neu-targeted vaccine that significantly reduced pulmonary metastatic burden and prolonged lifespan in canine osteosarcoma models, indicating potential for translation to human HER2-positive osteosarcoma. In pediatric osteosarcoma, 71% of patients exhibited immune reactivity to the anti-idiotypic vaccine 105AD7, with an absence of severe toxicities (82). Further clinical evidence by Ullenhag et al. (83) confirmed that 105AD7 vaccination induced robust T cell responses and facilitated recognition of CD55, an antigen structurally akin to the immunogen. Meanwhile, peptide vaccines targeting broadly expressed TAAs, including those associated with papillomaviruses and tumor rejection antigens, are under investigation for osteosarcoma and potentially other malignancies (84). Dendritic cells (DCs), due to their superior antigen presentation capabilities, are integral to initiating cytotoxic T lymphocyte (CTL) responses (85). Among immune-based interventions, DC-centric vaccines have gained attention, especially in refractory tumors such as osteosarcoma. Mackall et al. (86) revealed that metastatic or relapsed Ewing sarcoma patients achieved prolonged survival and minimal toxicity after receiving a combined immunotherapeutic regimen incorporating DC vaccines, autologous T cells, and influenza immunization—even under the constraints of chemotherapy-induced immunosuppression. Nonetheless, the efficacy of tumor-targeted vaccines in solid tumors, including osteosarcoma, remains limited. For example, a phase I trial combining decitabine with DC-based vaccination in patients with sarcoma or neuroblastoma resulted in complete remission in only 10% of cases, while 60% experienced disease progression (86). Despite these challenges, DC-based immunotherapies continue to offer promise, and future studies are warranted to optimize their efficacy, either as standalone treatments or in synergistic combination with other immune-modulating agents. Recent study showed that combining anti-CTLA-4 with CD103^+^ cDC1 dendritic cell vaccine therapy increased cDC vaccine efficacy against osteosarcoma lung metastases (87).

### 3.5 CAR-T therapy

Chimeric antigen receptor (CAR)-modified T cells have revolutionized adoptive immunotherapy, particularly in hematologic cancers, where they have demonstrated striking clinical benefits. In a pivotal study, 80% of patients with refractory or relapsed B-cell acute lymphoblastic leukemia achieved remission following infusion with CD22-directed CAR-T cells (88). Likewise, CAR-T therapies targeting CD19 have yielded response rates nearing 90% across various B-cell malignancies, culminating in the first FDA approval for a genetically engineered cell therapy (89). Tandem CD19/CD22 CAR T-cell therapy demonstrates superior efficacy compared to monovalent CD19 CAR T-cell therapy (90). Efforts have also been made to translate CAR-T approaches to osteosarcoma by targeting tumor-specific antigens. For instance, the anti-IGF-1R monoclonal antibody Cixutumumab failed to show clinical efficacy in a phase II trial (91). Additionally, HER2-targeted CAR-T therapy produced only a transient partial response in a patient with metastatic HER2-positive sarcoma (NCT00902044) (92). This diminished effectiveness is likely attributable to the pronounced molecular heterogeneity and complex genomic landscape of osteosarcoma, which undermines the utility of single-target strategies (93). Recently, a Phase I clinical trial (NCT02107963) evaluated the feasibility and safety of administering GD2-targeted CAR-T cells in pediatric and young adult patients with OS and neuroblastoma (94). Restoration of CAR-T cell functionality has been pursued via PD-1 blockade or genetic engineering to render CAR constructs insensitive to PD-1–mediated inhibition (95). Integrating CAR-T therapy with low-dose chemotherapy to attenuate immune suppression and reduce PD-L1 expression is ongoing (NCT04433221). The development of advanced CAR platforms capable of simultaneously targeting multiple antigens aims to mitigate relapse driven by immune escape mechanisms (96). Importantly, co-expression of PD-1 and TIM-3 on CD^8^ T cells mark a deeper state of exhaustion than PD-1 expression alone, implicating dual immune checkpoint blockade as a potentially more effective approach (97). Moreover, the co-engagement of PD-1 and TIM-3 results in the recruitment of SHP2 phosphatase to their intracellular ITIM and ITSM motifs, which collectively dampens TCR signaling, suppresses granzyme B production, and drives terminal T cell dysfunction (97, 98).

Despite the intrinsically aggressive and metastatic nature of osteosarcoma, rationally designed immunotherapies offer an avenue for clinical advancement. Nevertheless, the low immunogenicity of osteosarcoma, coupled with its elevated mutational burden and immunologically complex TME, necessitates integrative, multi-modal treatment approach (99, 100). Studies at MD Anderson Cancer Center have identified resistance mechanisms, including insufficient effector T cell infiltration, limited neoantigen presentation, and dominance of immunosuppressive pathways (101). Leveraging single-cell RNA sequencing, Cillo et al. (102) revealed enrichment of pro-inflammatory FABP4^+^ macrophages in pulmonary metastases and a depletion of osteoclasts in recurrent and chondroblastic subtypes. CD8^+^ T cells in these contexts exhibited elevated inhibitory receptor expression, suggesting potential benefit from checkpoint blockade during relapse. Further integrative transcriptomic analyses have illuminated the immunoregulatory functions of myeloid-derived populations—particularly mature regulatory dendritic cells (mregDCs)—which actively shape TME composition through intricate interactions with stromal and immune constituents (103). Advancing the field necessitates a deeper understanding of resistance mechanisms and the discovery of reliable predictive biomarkers, which will be instrumental in crafting rational, combinatorial immunotherapeutic regimens. While substantial hurdles remain, immune-oriented approaches hold promise for fundamentally altering the therapeutic trajectory of osteosarcoma.

## 4 Conclusion

Osteosarcoma remains a formidable clinical challenge due to its intrinsic aggressiveness, high metastatic potential, and profoundly immunosuppressive tumor microenvironment. While conventional therapies have plateaued in efficacy, the advent of immunotherapeutic strategies—including immune checkpoint inhibitors, tumor vaccines, CAR-T cells, and adoptive TIL therapies—has catalyzed renewed interest in reshaping the therapeutic landscape. Preclinical and early-phase clinical studies underscore the immunogenic potential of osteosarcoma, yet the limited success of monotherapies highlights the necessity for combinatorial and patient-tailored approaches.

Advancing immunotherapy in osteosarcoma will require a deeper mechanistic understanding of resistance pathways, immune evasion, and intratumoral heterogeneity. Emerging technologies such as single-cell transcriptomics, spatial profiling, and multi-omics integration offer valuable insights into immune cell dysfunction, myeloid cell plasticity, and stromal-immune crosstalk. Future efforts should prioritize the identification of predictive biomarkers, the rational design of synergistic regimens, and the stratification of patients for optimized immunotherapeutic benefit. Ultimately, immune-based interventions hold transformative potential to overcome current therapeutic bottlenecks and improve long-term outcomes in osteosarcoma.
